# Determinants of Mean Blood Pressure and Hypertension among Workers in West Africa

**DOI:** 10.1155/2016/3192149

**Published:** 2016-02-02

**Authors:** William K. Bosu

**Affiliations:** Department of Epidemics and Disease Control, West African Health Organisation, 01 BP 153, Bobo-Dioulasso 01, Burkina Faso

## Abstract

*Background*. This review was undertaken to estimate the mean blood pressure and evaluate its determinants as well as the determinants of hypertension among workers in West Africa.* Methods*. In a follow-up to an earlier study, a systematic search for articles published between 1980 and August 2015 was undertaken using major databases.* Results*. A total of 55 articles involving 34,919 different cadres of workers from six countries were retrieved. The mean systolic blood pressure (BP) ranged from 116.6 ± 1.3 mmHg to 151.7 ± 13.6 mmHg while the mean diastolic BP ranged from 69.6 ± 11.0 mmHg to 97.1 ± 9.1 mmHg. Population-wide prehypertension was common. The major determinants of mean BP and hypertension were similar and included male sex, older age group, higher socioeconomic status, obesity, alcohol consumption, plasma glucose, and sodium excretion. Ethnicity and educational level were inconsistently associated with hypertension. Workers at higher risk of cardiovascular event did not perceive themselves as such.* Conclusion*. The prevailing mean prehypertensive BP, low perception of risk, and clustering of risk factors call for interventions such as healthy diets, improved physical activity, and a favourable work environment. Successful models for improving the cardiovascular health of sedentary informal sector workers in Africa are urgently needed.

## 1. Introduction

The major modifiable risk factors for noncommunicable diseases (NCDs) are well known and have been the focus of the current WHO Plan of Action 2013–2020 under a 4-by-4 strategy [1]. Physical inactivity, unhealthy diet, smoking, and harmful alcohol use have been associated with increased all-cause, cancer- and cardiovascular-related mortality [2]. Consequently, the adherence to lifestyle factors is associated with as much as 66% reduction in all-cause mortality [3]. Applying such knowledge, often derived from studies in western countries, in policy and programmes for improved public health in resource-poor settings often requires customized approaches that take account of cultural appropriateness, cost-effectiveness, implementation capacity, and equity gaps [4].

The Sixtieth World Health Assembly, in endorsing the global plan of action 2008–2017 for workers' health, urged countries to institute workplace health programmes to promote health and to prevent NCDs, in particular through healthy dietary lifestyles and physical activity among workers [5]. Workers in West Africa deserve the best health and quality of lives during their productive working lives as well as in retirement. Concerted action should therefore be taken to adapt and implement the global plan of action for workers health. West African workers report multiple NCDs during their late working lives [6].

While the workplace health programmes are cost-effective in reducing NCDs [7], relatively little is known about the disease burden and existing programmes among workers in Africa. A recent systematic review among workers in West Africa reported a prevalence of hypertension of 12%–69% among workers [8]. This paper focuses on the factors and the determinants that are associated with blood pressure levels and hypertension among workers in the Economic Community of West African States (ECOWAS) Region. Knowledge of these determinants could enable better targeting of interventions to reduce elevated blood pressure among workers.

## 2. Methods

The study setting, detailed search strategy, data extraction, and definition of variables have been described elsewhere [8]. Briefly, the PubMed, Embase, and Google Scholar databases were systematically searched to identify articles estimating the prevalence of adult hypertension among workers in West Africa. In the original review, 45 papers published over the period from 1980 to September 2014 were retrieved. In this review, the publication date was extended to end of August 2015 using saved search items. The search terms were “hypertension” and “workers” combined in its various forms with the individual countries and collectively with West Africa. The bibliography of retrieved papers was screened manually to identify additional papers.

The inclusion criteria were that the total number of respondents was at least 75 and the minimum age of the workers 15 years. Population- or hospital-based studies on workers were excluded. Among the data extracted were the demographic characteristics of the workers, sampling technique, method of measuring the blood pressure (BP), prevalence of hypertension, mean systolic and diastolic BP (SBP and DBP), and the crude and adjusted factors associated with the mean BP or hypertension. Unlike the previous study that focussed on the prevalence, the present review focusses on factors and determinants associated with mean BP and hypertension.

## 3. Results

**Figure 1:**
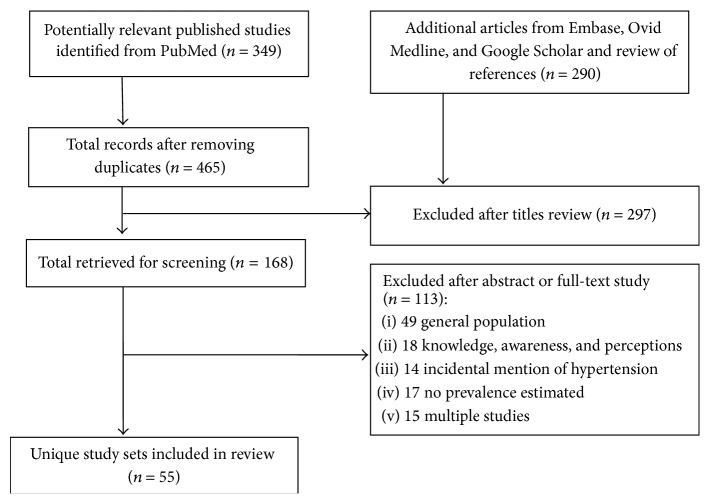
The process of selecting articles.

A total of 55 articles from six of the 15 ECOWAS countries satisfying the inclusion criteria were retrieved after screening 168 articles (Figure 1). Of the included articles, 41 (74.5%) were conducted in Nigeria, five conducted in Ghana, three each in Senegal and Togo, two in Cote d'Ivoire, and one in Liberia. The group of workers who were most frequently studied were civil servants [9–19], university workers [20–26], health care workers [27–32], traders [33–37], factory workers [38–40], farmers [41, 42], bankers [43–46], and office workers [47–52]. There were also studies on garage workers [53], music dealers [54], mill operators [55], port workers [56], drivers [57], and motorcyclists [58]. Except for three studies, all the studies were undertaken in urban or mixed settings. Overall, 34,919 workers were studies across the studies with a median sample size of 380 (range 75–5,200). Eight studies were conducted exclusively among male [39, 53, 57, 59–61] or female workers [33, 35]. The mean age of the workers across the studies ranged from 23.1 years in commercial cyclists to 63.0 years in rural farmers. In 51 of the studies in which hypertension was defined based on the 140/90 mmHg threshold, 6,745 workers were diagnosed with hypertension.

### 3.1. Mean Blood Pressure

Among 27 studies with information, the mean SBP ranged from 116.6 ± 1.3 mmHg among traders in Ijebu Ode, Nigeria [37], to 151.7 ± 13.6 mmHg among workers of a private telecommunication company in Senegal [47]. In one (3.7%) of these studies, the mean SBP was ≥140.0 mmHg; in five (18.5%), it was 130.0–139.9 mmHg; in 18 (66.7%), it was 120.0–129.9 mmHg; and in three (11.1%), it was <120.0 mmHg. The mean DBP ranged from 69.6 ± 11.0 mmHg among male factory workers in Nigeria [39] to 97.1 ± 9.1 mmHg among telecommunication workers in Senegal [47]. In one (3.7%) of the studies, the mean DBP was ≥90.0 mmHg; in two (7.4%), it was 85.0–89.9 mmHg; in eleven (40.7%), it was 80.0–84.9 mmHg; and in thirteen (48.1%), it was <80.0 mmHg.

### 3.2. Factors Associated with Hypertension and Mean Blood Pressure Level

#### 3.2.1. Biological Factors

Age was a ubiquitous risk factor present among the same group of workers such as civil servants [9–11, 17, 59], health workers [13, 15, 27, 29], or university workers [23, 26] or among different groups of workers [38, 49].

SBP, DBP, and hypertension increased with increasing age in both sexes in different geographical settings [9–12, 14, 29, 39, 42, 44, 49, 59, 62]. The change in the mean BP with increasing age was, however, minimal in two studies in Nigeria [39] and Liberia [41].

Hypertension was more frequent among males than among females in 22 of 27 studies in which they could be compared, using the 140/90 mmHg threshold definition. It was only in six studies that the prevalence was higher in females. In one study with results in both directions, the prevalence was higher among male bankers and female traffic wardens [46]. The prevalence of hypertension ranged from 11.2% to 68.9% in male workers compared with 0% to 43.5% in female workers. Similarly, SBP and DBP were consistently higher in men than in women in all age groups in most studies (Table 1) [9, 11, 38, 44, 49, 63].

The relationship between positive family history of hypertension and hypertension was rarely investigated. It was more common in nonhypertensives than in hypertensives [31, 46] with the difference being statistically significant in some studies [31]. After stratifying civil servants into two age groups (<40 years or ≥40 years), the prevalence of hypertension was found to be higher in those with a positive family history of hypertension than in those without, but the difference was not statistically significant [13].

Ethnicity was inconsistently associated with hypertension in some studies. Giles et al. [41], in a large study in rural Liberia, found statistically significant differences in age- and sex-adjusted SBP and DBP by ethnicity. The prevalence ranged from 5% among the Grebo ethnic group to 24% among the Mano ethnic group. However, there were no statistically significant differences in the prevalence of hypertension by ethnic origin in Nigeria [11].

In Calabar, the Efik ethnic group had a significantly higher SBP and DBP as well as a higher prevalence of hypertension than the other ethnic groups [12]. Male civil servants in Sokoto from the southern and eastern tribes of Nigeria were reported to have significantly higher diastolic BP than those from the northern tribes [10]. However, these differences in the DBP disappeared after adjusting for age and BMI. Similarly, in Dakar, male workers of the Peul ethnic group had significantly higher systolic blood pressure levels than those of the Serer group but the difference disappeared in multivariate analysis [38].

#### 3.2.2. Obesity

The prevalence of obesity among the workers, as provided by 23 studies, ranged from 2.0% among automobile garage workers in Kumasi, Ghana [53], to 42.1% among health workers in Umuahia, Nigeria [29]. The prevalence of overweight or obesity, as reported by 16 studies, ranged from 25.6% among pharmaceutical industry workers [40] to 97.7% among Senegalese workers of an information technology (IT) company [51]. It was more than 50% among civil servants [9, 13], health workers [27, 31], traders [35, 37], medical school lecturers [22], traffic wardens [46], and IT company workers [51].

**Figure 2:**
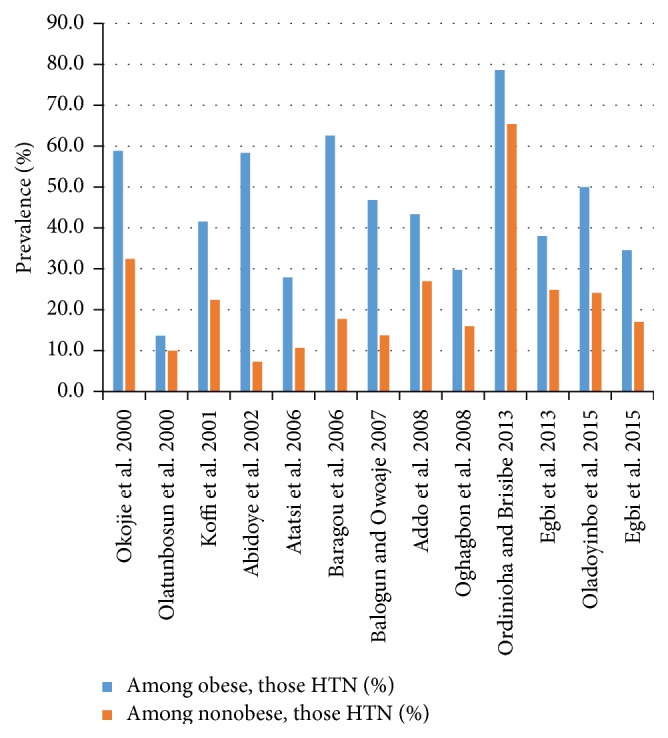
Prevalence of hypertension (HTN) in obese and nonobese workers.

Obesity was one of the strongest independent risk factors associated with hypertension. In 14 studies in which the prevalence of hypertension could be evaluated for obese and nonobese workers, the prevalence ratio ranged from 1.2 to 8.0. In most cases, the differences were statistically significant. The prevalence of hypertension ranged from 27.9% to 78.6% among obese subjects compared with 7.3% to 65.4% in nonobese subjects (Figure 2). Among health workers in a university teaching hospital, the odds ratio (OR) of the association between obesity and hypertension was 2.12, *p* = 0.004 [27]. Civil servants in Kaduna younger than 40 years and who were overweight or obese were five times as likely as those with healthy weight to have hypertension [13].

As with hypertension, fat-related variables such as BMI, waist circumference, and waist-to-hip ratio were frequently associated with BP [10]. The mean BP was positively correlated with BMI in all studies with available information [10, 15, 37–39, 44, 62, 64]. Ogunlesi et al. [39] found a correlation coefficient of 0.22 (*p* < 0.01) between BMI and BP. The correlation between BP and the BMI may not be linear. One study reported that, below the threshold of 21.5 kg/m^2^, BP was not correlated with BMI [62].

Despite being a strong risk factor for hypertension and mean blood pressure, overweight and obesity were not well appreciated among workers. Among university health workers in Ghana who did not consider themselves overweight, nearly half were actually obese (11%) or overweight (37%) actually [23]. Similarly, only 29.0% of university teaching hospital health workers who were overweight or obese considered themselves to be overweight [27].

#### 3.2.3. Physical Activity

The prevalence of hypertension was significantly higher among those with low physical activity compared with those with moderate or intense physical activity [9]. Workers whose jobs were physically demanding such as automobile workers [53], mill operators [55], and plantation workers [41] appeared to have a lower prevalence of hypertension than those whose jobs were largely sedentary such as traders [35, 66], office executives [47, 49], and civil servants [9, 11, 16].

In Maiduguri, Nigeria, the mean SBP was significantly and inversely associated with four levels of physical activity [19]. Civil servants and health workers who were highly sedentary had a mean BP that was 19.1 mmHg higher than those who were highly physically active. The BP ranged from 123.8 ± 13.8 mmHg in highly active workers to 142.9 ± 18.7 mmHg in highly sedentary workers. Moderate-to-vigorous physical activity was significantly and negatively correlated with BMI and waist circumference, as well as SBP (*r* = −0.224, *p* < 0.001) and DBP (*r* = −0.194, *p* < 0.001).

#### 3.2.4. Alcohol Consumption

Hypertension was significantly more frequent in alcohol drinkers than in abstainers [11]. Kadiri et al. [44] found that regular and moderate drinking and not just mere drinking was associated with hypertension. The prevalence was higher in medium and heavy drinkers than in nondrinkers and light drinkers [12]. It was also higher among medium and heavy smokers. University College of Health Sciences staff in Ghana who were hypertensive had six times the odds of consuming alcohol four or more times weekly compared with those who are not hypertensive (OR 6.0, 95% CI 1.5–23.8) [23]. In Dakar, neither alcohol nor tea consumption was significantly associated with blood pressure levels [38].

#### 3.2.5. Sodium Excretion

Using the 140/90 mmHg threshold, sodium excretion was higher among female hypertensives than in normotensives but this was not the case in men [10]. However, using the higher 160/95 mmHg threshold, both sodium and potassium excretion were significantly higher in male and female hypertensives compared with normotensives.

The 24-hour urinary sodium excretion and the sodium : potassium ratio were weakly but significantly correlated with SBP and DBP among a combined group of railway workers, urban poor informal sector workers, and rural farmers aged 45 years and over in Oyo State [60]. Urinary potassium excretion was neither correlated with SBP nor DBP. Among the male rural farmers in this set, both the 24-hour sodium excretion and sodium : potassium ratio correlated strongly and significantly with both SBP and DBP (*r* = 0.32–0.51). In contrast, overnight urinary sodium, potassium or creatinine excretion, and sodium-potassium, sodium-creatinine, or potassium-creatinine ratio were not significantly correlated with SBP or DBP in men or women in Sokoto [10]. Neither SBP nor DBP was significantly correlated with serum creatinine or glomerular filtration rate in the civil servants of Bayelsa State [15].

#### 3.2.6. Diabetes

The strongest risk factors associated with hypertension in telecommunication workers in Dakar were diabetes (OR 3.9, 95% CI 2.2–6.9), age ≥ 40 years compared with age <40 years (OR 2.8, 95% CI 2.2–3.6), overweight (OR 2.2, 95% CI 1.8–2.8), and male sex (OR 1.6, 95% CI 1.3–2.1) [38]. Diabetic bankers in Lagos, Nigeria, were 4.3 times as likely as those without diabetes to have diastolic hypertension [43].

Egbi et al. [15] observed that, unlike DBP, SBP was significantly correlated with random blood glucose (RBG) and serum urea in civil servants in Bayelsa State. However, the correlation was weak with coefficients of 0.13 and 0.18, respectively, unlike in civil servants where the same group of investigators found that DBP was also significantly correlated with RBG in health workers [31].

#### 3.2.7. Socioeconomic Factors

Socioeconomic status was assessed using salary grades or, as a proxy, staff ranking or educational attainment. Hypertension was more common in workers with higher salary grades than in those with lower salary grades [12, 18]. It was more common in senior staff than in junior staff [9, 23, 24, 67], even after adjusting for age [10, 11]. The age-adjusted prevalence in senior and junior staff was 43% versus 23%, respectively, among male civil servants in Benin City [11]. The differential remained after controlling for BMI. The prevalence of hypertension among the male senior staff within the highest tertile band of BMI (23.8–42.2 kg/m^2^) was significantly higher than that among the junior staff (61.4% versus 37.5%, *p* = 0.005).

In Sokoto, the age-adjusted prevalence of hypertension in senior (19.3%) and junior (19.8%) staff was similar [10]. However, senior staff were observed to have three times the prevalence of junior staff when the higher BP 160/95 mmHg cut-off limit was used (11.2% versus 3.6%).

Among civil servants in Accra in 2006, the differences in the prevalence of hypertension among the different employment grades were statistically significant, with senior staff more likely to be hypertensive than junior staff though the direction was not entirely uniform [9]. The prevalence was 20.8% among unskilled workers, 30.1% among clerical staff, and 37.4% among professional staff but only 11.7% among the directors. In Abidjan, the prevalence of hypertension in medical specialists was 2.5–3.5 times that among doctors, nurses, and nurse auxiliaries [28]. Consistent with these observations, Kaufman et al. [60] found that the prevalence increased across the socioeconomic gradient from rural farmer to urban poor to railway workers: 14.0%, 25.0%, and 29.0%, respectively, in Oyo State in 1994. However, Bunker et al. [10] observed similar age-adjusted prevalence of hypertension among senior and junior grade civil servants in Sokoto.

Generally, as with hypertension, the mean BP was higher in senior than in junior staff [11, 52]. An exception was in the Sokoto study in which junior civil servants had higher SBP but lower DBP after adjusting for age and BMI [10]. Unlike DBP, the mean SBP increased along the gradient from junior staff through senior staff to management among workers in a media organization in Ghana [52]. However, in a few studies, junior staff had significantly higher BP than senior staff [10, 38].

The association between educational level of workers and hypertension or mean BP levels was inconsistent. A higher prevalence of hypertension was reported in those with low level of education in some studies [13, 31, 38, 65] as well as in those with higher levels of education [11, 39]. The pattern at the different levels of education was downward [65], upward [39], or U-shaped with the highest prevalence in those with the lowest and highest levels of education [11]. In Dakar, the age-adjusted prevalence was three times as high in illiterates as in those with primary or secondary education, with the difference being statistically significant [38]. The prevalence of hypertension increased with increasing educational level among factory workers in Ibadan, Nigeria [39]. The pattern remained after adjustment for age, BMI, pulse rate, alcohol drinking, and antihypertensive treatment. Those with moderate and highest levels of education were 2.0 and 2.4 times as likely as those with the lowest levels of education to have hypertension, with the differences being statistically significant.

Among male factory workers in Ibadan in 1994, the mean age-adjusted SBP and DBP increased with increasing years of education [39]. In those with low level of education (0–9 years), the mean SBP and DBP were 125.8 mmHg and 67.9 mmHg, respectively, while, in those with high level of education (≥13 years), they were 130.1 mmHg and 71.7 mmHg, respectively. The pattern persisted after adjusting for age, BMI, pulse rate, alcohol drinking, and antihypertensive treatment. BP also correlated with years of education in male bank workers in Ibadan, but not in females [44]. Unlike the male factory workers, the age-adjusted SBP and DBP in male bankers were highest in those with secondary education. In women, the highest BP and lowest BP were in those with primary and secondary education, respectively. In Dakar, the age-adjusted SBP and DBP were highest in illiterates and lowest in those with primary education, with the difference being statistically significant [38].

#### 3.2.8. Urban Residence

The prevalence of hypertension was higher in urban than in rural areas. The prevalence of hypertension in rural settings [41, 42, 60] ranged from 12.3% to 13.9% and was much lower than that in urban settings (12.0%–60.2%). Compared to other similarly aged workers in a broadly common environment, Kaufman et al. found that the prevalence of hypertension in urban poor informal sector workers was 1.8 times that of rural farmers [60].

As with hypertension, the mean SBP and DBP were higher in urban than in rural settings [14]. The mean SBP and DBP levels in men aged ≥45 years and older in Oyo State in Nigeria followed an urban-rural and socioeconomic gradient. They were highest among urban railway workers and lowest in rural farmers with the levels in the urban poor informal sector workers being intermediate [60]. The length of residence in urban area was, however, not associated with BP among workers in Dakar [38].

#### 3.2.9. Occupational Exposures

In the earliest study covered by this review, rural office workers had a higher mean BP than rural field labourers in the Bendel State of Nigeria in 1976 [68]. Idahosa [59] observed that male civil servants were significantly more likely to have hypertension than the more physically active group of workers, policemen, in Benin City (33.6% versus 24.4%; *p* < 0.001). However, difference between the two groups ceased to be statistically significant when the higher BP cut-off point of 160/95 mmHg was used.

Female industry workers in Dakar exposed to noise levels that made it impossible to converse at the workplace were more likely than those not exposed to be hypertensive, but the difference was not statistically significant [38]. Similarly, Ebare et al. [54] found that, although mean SBP and DBP increased with increasing exposure across three categories of noise levels among music dealers in Benin City, the differences were not statistically significant. However, the prevalence of hypertension increased significantly with exposure to increasing noise levels (<85 dB = 14.3%, 85–90 dB = 39.5%, and >90 dB = 55.6%; *p* = 0.003).

Investigators in the study of civil servants in Benin City confirmed their hypothesis that hypertensives experience larger increases in BP during behavioural stress than normotensives [67]. They also found that, even when men and women occupied similar status jobs (senior staff or junior staff), men still had greater changes in their BP in response to stress than women. Similarly, Owolabi et al. [32] found that health workers who were classified as being exposed to high levels of work-related stress (based on a job-demand control questionnaire) were significantly more likely to have hypertension than others exposed to lower levels of stress (42.4% versus 12.1%, *p* < 0.001).

In terms of the relationship between working conditions and mean blood pressure, the age-adjusted SBP and DBP were higher among male shift workers in Dakar than in those with regular work schedule [38]. The age-adjusted BP did not differ between women exposed to occupational noise compared to those not exposed. The travel distance to work and occupational or recreational physical activity were not related to BP.

#### 3.2.10. Other Factors

Hypertension was associated with obstructive sleep apnoea among the university staff in Ogbomosho, Oyo State, Nigeria (OR 3.2) [69]. Knowledge about risk factors associated with hypertension was not significantly associated with a lower prevalence. In a study comparing the knowledge of risk factors among bankers and traffic wardens in Ilorin, the former were more likely to know five risk factors. Yet, the bankers, being of a higher socioeconomic class and having a different risk factor profile, had a higher prevalence of hypertension than the traffic wardens (34.4% versus 22.2%; *p* < 0.05) [46]. Neither coffee consumption nor the daily smoking of two to five sticks of cigarettes was associated with hypertension among university workers in Ile Ife [26]. However, parity of 4 or more children was significantly associated with hypertension in these workers. In contrast, age-adjusted parity in women was not significantly related to BP in Dakar [38].

In Ghana, 31.7% of media workers self-reported having hypertension [52]. However, on screening, twice as many workers (60.2%) were found to have diastolic hypertension. The greatest gap was observed among junior staff in whom 3.6 times as many as those self-reporting hypertension were identified as having diastolic hypertension.

#### 3.2.11. Clustering of Risk Factors

There was clustering of cardiovascular risk factors [25, 47, 51, 70]. Out of 1,229 telecommunication workers screened in Senegal, 341 (27.7%) had one cardiovascular risk factor, 632 (51.4%) had two risk factors, and 256 (20.8%) had three or more risk factors [47]. Seven percent of those diagnosed with hypertension had a moderate Framingham risk score while 18% were found to be at a high risk of a cardiovascular event. Nearly three-quarters of hypertensive telecommunication workers in Lome were assessed to have high (32.0%) or very high (41.8%) risk of cardiovascular event [71].

Among 525 university staff in Ibadan, Nigeria, 67.4% reported one risk behaviour, while 30.0% reported two or more risk behaviours (unhealthy diet, sedentary living, excessive alcohol use, and smoking) [25]. Remarkably, those with multiple risk factors did not perceive themselves to be at any greater risk of NCD morbidity than those without. Similarly, among the staff of another university in Ogbomosho, Nigeria, 96 male staff had average of 2.6 cardiovascular risk factors compared with 3.3 risk factors among 110 females [20]. The risk factors evaluated were smoking, hypertension, impaired glucose tolerance, obesity, elevated triglyceride level, elevated low-density lipoprotein- (LDL-) cholesterol, low level of high-density lipoprotein- (HDL-) cholesterol, elevated total cholesterol (TC), and presence of left ventricular hypertrophy (LVH) on electrocardiography (ECG).

#### 3.2.12. Summary

In sum, risk factors that were positively associated with hypertension and increased BP in bivariate analysis included older age group, male sex, urban residence, higher socioeconomic class, physical inactivity, obesity, alcohol consumption, sodium excretion, and sodium-potassium ratio. High levels of work-related stress were also related to hypertension while shift work was related to mean BP. Unlike blood pressure, the exposure to high noise levels was associated with hypertension. The relationship between educational attainment and ethnicity and hypertension was inconsistent.

### 3.3. Determinants of Hypertension and Mean Blood Pressure

Seven studies evaluated the determinants of blood pressure levels while nine evaluated the determinants of hypertension among workers such as civil servants, health workers, and bank workers using logistic or multiple linear regression. Three studies examined determinants of both blood pressure and hypertension [10, 11, 18]. Generally, the studies rarely included more than five variables in their modelling (Tables 2 and 3).

Among male civil servants in Benin City in 1987-1988, age, BMI, alcohol drinking, and senior staff grade were independent risk factors for hypertension or BP [64]. In females, only BMI was independently associated with BP or hypertension (OR 13.2, comparing third tertile BMI with first tertile). Education was not independently associated with hypertension in men [11]. The strongest determinants of hypertension in men were staff grade, BMI, and alcohol consumption (Table 3).

In the 1990 Sokoto Civil Servants Study, the age- and BMI-adjusted DBP in male civil servants was significantly higher in senior staff than in junior staff (75.7 mmHg versus 73.3 mmHg, *p* < 0.01) [10]. In contrast, the adjusted SBP was lower in senior staff than in junior staff, but difference was not statistically significant. Age group (10-year intervals) and BMI tertile in men and sodium excretion in women were independently related to hypertension based on the 140/90 mmHg threshold [10]. Sodium excretion and senior staff grade became additional risk factors for hypertension in men when the model used the higher threshold 160/95 mmHg as the outcome.

In Ibadan, age, male sex, BMI, and plasma glucose were significantly associated with SBP in multivariate ANOVA model among 856 male and female civil servants [18]. Among only men, age, BMI, and glucose were highly significant while, in women, only age and BMI were significantly associated with SBP. In the model with DBP as outcome, age, sex, and BMI but not plasma glucose were significant factors in male and female civil servants combined. In the separate models for male and female civil servants, age and BMI were significant predictors of DBP.

Similarly, age, plasma glucose, and waist-to-hip ratio (WHR), as well as a family history of diabetes were the predictors of hypertension among the same group of civil servants in Ibadan [18]. The strongest predictor was WHR (OR 1.35). In a separate logistic regression model, height was found to be associated with abnormal glucose tolerance but not hypertension [72].

In addition to male sex, increasing age, and weight, marital status, number of children in the family, higher salary scale, and tobacco consumption were independently associated with SBP in a large study involving 5,200 civil servants and factory and plantation workers in Calabar [12]. Only the number of children in the family and salary scale were associated with DBP (Table 2). Occupation, educational level, support system and height were inversely associated with SBP.

Kaufman et al. [60], on the other hand, found that the number of children was significantly and strongly associated with a lower SBP among women in the Oyo State in 1994. Among rural farmers, pulse rate and sodium-potassium ratio were positively associated with SBP while BMI was positively associated with DBP. The strongest risk factor for increased SBP or DBP in either men or women was age ≥ 55 years while nonmigrant status (participants born in the same community in which they currently live) was significantly associated with lower BP.

Ogunlesi et al. [39] found that, among male factory workers, education level was significantly associated with BP, after controlling for age, body mass index, pulse, and alcohol consumption. The multivariable-adjusted mean SBP and DBP were each about 4 mmHg higher in those with the highest education level compared to those with the lowest educational level (*p* < 0.05). Male factory workers with 13 or more years of education were two and half times as likely to have hypertension as those with less than 10 years of education, after adjusting for age, body mass index, pulse, and alcohol consumption (OR 2.4, 95% CI 1.2–5.1) [39].

Among bank workers in Ibadan, age- and BMI-adjusted BP were correlated to years of education and income in men, but not in women [44]. However, there was no consistent association between the mean SBP and DBP and educational level in men and women. The highest mean SBP and DBP were not found among male or female subjects with tertiary education. Rather, in men, they were found in those with secondary education while, in women, they were found among those with primary education. Conversely, the lowest mean BP was found in men with and women with secondary education.

In multiple linear regression analyses, Lang et al. [38] found that, among 914 male industry and hotel workers in Dakar, increasing age, number of glasses of tea consumed daily, and illiteracy were significantly associated with SBP. However, only age and BMI were significantly associated with DBP. Shift work was associated with neither SBP nor DBP. Ethnicity was significantly associated with SBP (*p* = 0.05) but not with DBP.

The determinants of hypertension among civil servants in Kaduna in 2012 were older age and a positive family history of hypertension [13]. Those who were aged ≥ 40 years were 6.7 times (95% CI 4.1–11.0) as likely as those aged < 40 years to have hypertension. Those with a positive family history were 1.5 times (95% CI 1.1–2.1) as likely as those without a family history to have hypertension.

In the Accra Civil servants Study, Addo et al. [9] found that age groups older than 35 years, male sex, and obesity were the determinants of hypertension in a model adjusted for age and sex. However, after adjusting for additional variables (BMI, physical activity, and alcohol use), obesity lost its statistical significance. Alcohol intake was not related to hypertension in all the models while moderate and intense physical activities were protective. Multiple regression analyses showed that BMI, waist circumference, and SBP were inversely associated with physical activity among civil servants and health workers in Maiduguri, Nigeria [19].

Age, alcohol, and BMI were the predictors of hypertension among market workers in Enugu [36]. Sex, smoking, snuff tobacco, and WHR were not significantly associated with hypertension. Among health workers in a tertiary hospital in Jos City, Nigeria, alcohol (OR 2.58, *p* = 0.03) and obesity (OR 3.37, *p* = 0.006) were independently associated with hypertension [27]. However, among health workers in a tertiary hospital in Yenagoa, Nigeria, truncal obesity (as determined by waist circumference) but not general obesity was independently associated with hypertension after adjusting for age, gender, marital status, education, family history of diabetes or hypertension, and behavioural risk factors [31]. Health workers who had abdominal obesity were 3.2 times as likely as those with normal waist circumference to have hypertension (95% CI 1.15–9.06). Besides waist circumference, age was the only other variable that was independently associated with hypertension, using health workers younger than 30 years as the reference group (Table 3).

#### 3.3.1. Summary

It can thus be concluded from these multivariate analyses that the major determinants of increased BP and hypertension among workers in West Africa were similar to the factors. They included older age group, male sex, higher income, senior staff grade, alcohol intake, BMI, general or truncal obesity, alcohol consumption, plasma glucose, sodium excretion, and sodium-potassium ratio. Nonmigrant status was associated with decreased BP while moderate-to-vigorous physical activity was protective against hypertension and BP. The number of children, educational level, and ethnicity were inconsistently associated with BP or hypertension.

## 4. Discussion

This is the first systematic review of the determinants of arterial blood pressure and hypertension among workers in West Africa. The review involved a large number of workers covering different groups of formal and informal sector workers, included articles in the French language, and covered a 35-year period. It showed that the mean BP levels among workers in West Africa are high. Twenty-three (85.1%) of the 27 studies reporting mean BP were in the prehypertension range. Nearly a fifth of the studies recorded mean populations' BP in the high-range prehypertension category. High mean BP is not unique to workers. A recent systematic review of 27 community-based studies in Nigeria including four covered by the current review estimated a pooled mean SBP of 128.6 mmHg (95% CI 125.5–130.8 mmHg) and a mean DBP of 80.6 mmHg (95% CI 78.5–82.7 mmHg) [73].

Prehypertension, particularly high-range grade, has been associated with increased risk of incident stroke and cardiovascular morbidity and mortality [74, 75]. Persons with prehypertension may have an increased risk of progressing to hypertension [76]. Consequently, the Seventh Joint National Committee recommended nonpharmacological measures in individuals such as weight management, reduced dietary intake of salt, physical activity, and moderation of salt intake to manage prehypertension [77]. Antihypertensives have also been shown to prevent the progression to hypertension in individuals with prehypertension [78]. There is a dearth of studies specifically evaluating the impact of population-based strategies on prehypertension. However, community-based interventions such as health education campaigns in the media, schools, and workplaces, nutritional advice, and worksite exercise breaks have been shown to reduce mean BP in low-income countries [79].

The factors and determinants associated with BP and hypertension were broadly similar. The most consistent biological factors were increasing age and male sex while the most consistent lifestyle factors were sodium excretion, alcohol consumption, and obesity. As in general population studies, workers in urban settings had higher BP than those in rural settings [73, 80, 81].

Among the lifestyle factors, dietary salt intake is one of the least studied in West Africa although reduction in dietary salt, fat, and sugar intake is one of the priority actions recommended by the expert Lancet NCD Group [82]. It is also one of the voluntary NCD targets in WHO's Global Action Plan [1]. In this review, urinary sodium excretion was one of the independent risk factors associated with hypertension. One-third of the relatively young population of bankers in Benin City reported that they added extra salt to their food [45]. In Nigeria, market workers spend most of their day at their market and have little options except to buy fast food or food from street vendors, which is typically high in salt content [36].

There are hardly any successful salt reduction programmes in Africa where, unlike in developed countries, salt tends to be added to the food during cooking and at serving and the consumption of processed foods is low [83]. In many countries, screening of food vendors is limited to microbiological safety with little interest in the nutritional quality of food that is sold to the general public including school children and workers. A community-based health promotion programme in 12 villages in Ghana led to modest declines in the BP after six months, although there was no significant change in the urinary sodium excretion. In the Iran Healthy Heart Program, an integrated set of interventions involving public education, healthy nutrition, physical activity, tobacco control, and stress management using the mass media, community participation was associated with statistically significant reductions in dietary salt intake, sodium excretion, and BP after a nine-year follow-up [84]. There are more concerted efforts by stakeholders including health professionals, regulatory agencies, industry, local government, social welfare, and the civil society to reduce dietary salt intake in West Africa.

Inadequate knowledge about hypertension in Africa is pervasive, with belief that it is due to fate, evil spirits, or excessive thinking and that it is curable [8, 20, 45, 85]. Some West African workers had a low perception of their risk of complications. Those who were obese or had multiple risk factors did not correctly identify themselves as such and so were little concerned about their risk profile. There was a significant gap between those who self-reported that they did not have hypertension and those who were found to have hypertension [52]. The misperceptions and the gaps in knowledge call for regular programmes to be instituted to educate formal and informal sector workers as well as the general public about cardiovascular health in the context of the current nutrition transition.

Over 90% of bankers in Nigeria felt that their work was stressful [45]. As with similar formal and sedentary jobs such as civil servants, bankers work long hours daily, up to six days weekly. Workers who constantly face this combination of sedentariness and stress should not simply be left to take care of themselves. As this review showed, there is often clustering of cardiovascular risk factors. Companies, therefore, have a responsibility to provide the enabling environment and programmes to encourage their workers to adopt healthy lifestyles within and without work hours. The example of an Employee Well-Being programme in Ghana involving eight private companies in partnership with the Ministry of Health and the German Cooperation is commendable [86]. The programme aims to improve the health, social protection, and financial wellness of employees and their immediate families. Worksite health programmes could improve cardiovascular health even at low attendance rates [87].

Obesity emerged as a strong predictor of BP and hypertension among workers. Successful models are needed for both formal and informal sector workers in resource-poor settings. In developing countries, community-based interventions have generally not been effective at reducing obesity. In a systematic review of community-based interventions [79], only one of four studies involving worksite interventions involving the integration of exercise breaks into organizational routine in Mexico was accompanied by reductions in BMI and waist circumference [88]. In western countries, successful worksite interventions for weight reduction combine diet, physical activity, and environmental changes [89]. Interventions such as price discounts for healthier foods from workplace canteens or vending machines and internet- or phone-based nutritional and exercise counselling, successful in western countries, may not be relevant or readily applicable in poor countries. On the other hand, health education to reduce fat, sugar, and alcohol and increase fruit and vegetable intake; small group sessions on increasing knowledge on weight management; newsletters, posters, and health promotional materials; and encouraging commuting to work, use of stairs, encouraging walking at lunch, environmental prompts to promote physical activity, and healthy eating and promoting group exercises could be useful.

The relationship between certain variables such as ethnicity and education among workers and hypertension in this review was not clear. Longitudinal studies are needed to better elucidate the determinants of incident cardiovascular disease in sub-Saharan Africa [90]. Studies are also needed to document what are the best delivery models to reduce chronic disease risks among sedentary workers in the informal sector with low literacy levels. Systematic reviews and meta-analysis to synthesize evidence on the prevalence or the determinants would help improve current understanding of the epidemiology of chronic diseases in Africa [73, 91–94].

Some limitations of this review should be noted. There was wide heterogeneity in the study population, definition of variables, methods of measurements, and analyses. These limit the generalizability of the findings to different types of workers in West Africa. Several studies adjusted for only a limited number of potential confounders and so may account for the differences in the effect sizes between studies. There was particular challenge in retrieving papers in this systematic review as 45% of the articles were published in less known African journals, some of which did not offer online full-text articles. The search strategy was meticulous and the database was comprehensive and so it is unlikely that significant studies among workers may have been missed. These limitations notwithstanding, the findings relating to factors and determinants were broadly consistent between studies conducted among workers and with studies conducted in the general population.

## 5. Conclusion

There are modifiable risk factors associated with BP and hypertension in workers in West Africa. Risk factors independently associated with mean BP include male sex, increasing age, marital status, higher income, senior staff grade, BMI, sodium excretion, and sodium-potassium ratio. The major determinants of hypertension among West African workers were similar to those of BP and included male sex, older age group, higher socioeconomic status, general or truncal obesity, alcohol consumption, plasma glucose, and sodium excretion. Measures to reduce mean BP and hypertension should include population-wide and worksite strategies to reduce dietary salt intake and other risk behaviours, reduce stress, improve physical activity, and increase knowledge of workers about cardiovascular health. More collaboration is needed between health sector, labour, local government, industry, nutrition and food societies, civil society, and workers' associations to prioritize the health of formal and informal sector workers in Africa.

## Figures and Tables

**Table 1:** Age-specific mean blood pressure among workers in West Africa.

Study population	Location	15–24			25–34			35–44			45–54			55–64		
		M	F	T	M	F	T	M	F	T	M	F	T	M	F	T
Mean systolic blood pressure																
Bank workers [44]	Ibadan	118.2 ± 14.2	111.0 ± 11.4		114.8 ± 14.9	110.7 ± 15.7		118.9 ± 17.2	117.6 ± 17.8		131.7 ± 20.7	124.0 ± 20.4		144.2 ± 14.3	110	
Civil servants [9]	Accra				125	113.5	119.0	127.5	117.5	125.0	135.5	130.8	134.0	140.3	131.8	137.0
Civil servants [11]	Bendel State	NA	NA	NA	123.1	114.2	nr	127.7	117	nr	135.7	128.6	nr			
Civil servants [10]	Sokoto	121.6	116.5		122.0	122.5		127.5	129.2		138.6					
Health care workers [29]	Umuahia, Abia State									124.49			128.39			135.00
Male factory workers [39]	Ibadan	127.2 ± 9.3			128.2 ± 12.4			126.7 ± 13.2			132.8 ± 21.1					
Rubber plantation workers [41]	Rural	127.3	120.0		125.0	121.4		124.9	125.3		126.8	135.2		127.7	144.85	
Senior executives of industries and companies [49]	Benin City, Edo State	NA	NA	NA	122.7	121.5	122.3	125.6	123.6	125.3	137.2	133.9	136.4	142.0	140.0	141.5

Mean diastolic blood pressure																
Bank workers [44]	Ibadan	73.1 ± 9.8	69.4 ± 8.5		73.8 ± 10.6	70.0 ± 11.1		76.7 ± 11.2	76.6 ± 11.7		85.0 ± 12.0	76.2 ± 10.8		87.5 ± 11.3	70.0	
Civil servants [9]	Accra				72.5	69.0	71.3	77.5	76	76.5	84.5	83.5	84.0	85	79.8	84.5
Civil servants [11]	Bendel State	NA	NA	NA	79.1	74.8	nr	82.3	74.8	nr	86.6	83	nr			
Civil servants [10]	Sokoto	67.6	69.6		71.1	75.1		76.3	78.5		82.6					
Health care workers [29]	Umuahia, Abia State									78.06			80.77			84.87
Male factory workers [39]	Ibadan	63.8 ± 9.7			68.3 ± 10.0			72.6 ± 10.6				75.4 ± 14.0				
Rubber plantation workers [41]	Rural	71.0	69.5		71.8	72.0		72.2	73.5		75.0	75.7		73.8	78.8	
Senior executives of industries and companies [49]	Benin City, Edo State				75.7	76.5		90.2	77.8		88.6	83.7		91.7	90.3	

^*∗*^Mean blood pressure ± standard deviation.

Bunker et al. 1996 [10]: lowest age groups 20–24 in both men and women. Only 3 age groups were reported for women; Giles: lowest age groups 20–24 in both men and women; last age group > 55 y; Uwanuruochi et al. 2013 [29]: lowest age groups 40–44.

Addo et al. 2008 [9]: median SBP and DBP values.

M: males; F: females; T: total sample.

**Table 2:** Determinants of arterial blood pressure in workers in West Africa.

Study population	Location	Determinants of BP	Age	Sex	Physical activity	Obesity or adiposity	Alcohol	SES	Others	Variables in model	Analysis
Civil servants [11]	Benin City	Age, BMI, alcohol consumption, and senior staff grade independently and positively associated with BP in men. In women, only BMI independently associated with BP.	BP related to age in senior staff but not in junior staff in males. BP not significantly related to age in females.	—	—	Correlation with SBP and DBP, *r* = 0.27 and 0.29 in male senior staff. In male junior staff, *r* = 0.25 and 0.26, respectively.	Alcohol drinking positively associated with BP in men but not in women.	Staff grade (senior staff) positively associated with BP in men but not in women.		Age, BMI, alcohol drinking, and staff grade.	Multiple regression

Civil servants [10]	Sokoto	Age- and BMI-adjusted DBP in male civil servants in the 25–54 age group significantly higher in senior staff than in junior staff (75.7 versus 73.3 mmHg, *p* < 0.01). In contrast, the adjusted SBP was lower in senior staff than in junior staff, but difference was not statistically significant.								Age, BMI.	ANOVA, logistic regression

Civil servants [18]	Ibadan	In both sexes combined, age, sex, and BMI were highly significant for both SBP and DBP. Further, plasma glucose predicted SBP but not DBP. In men and women, age and BMI were significantly associated with BP.	Age determinant for SBP and DBP in both sexes separately and combined.	When both sexes combined, male sex is a determinant	—	BMI predicts both SBP and DBP.		—	Plasma glucose, family history of diabetes.	BMI, glucose, age, and sex.	Multivariate ANOVA model

Civil servants, factory and plantation workers [12]	Calabar	Sex, age, marital status, number of children in the family, salary scale, tobacco consumption, and weight associated with SBP. Number of children in the family and salary scale associated with DBP. Occupation, educational level, support system, and height were protective of SBP.	Increasing age	Male sex		Weight		Salary scale for both SBP and DBP.	Marital status, parity, and tobacco use associated with SBP. Occupation, educational level, support system, and height were protective of SBP.	Sex, age, marital status, number of children, occupation, educational level, salary scale, social security, ethnicity, tobacco, alcohol, height, weight, SBP, and DBP.	Multiple linear regression

Retired railway workmen, rural farmers ≥ 45 years [60]	Idere village & Ibadan	BMI, waist circumference, radial pulse, and urinary sodium : potassium ratio were positively and significantly associated with blood pressure. Ambient temperature, nonmigrant status, and number of children were negatively associated with mean blood pressure.	Age ≥ 55 years strongly associated with SBP in men.		BMI positively and significantly associated with SBP and DBP in men, but not in women.				Nonmigrant status and ambient temperature are negative predictors while waist circumference, sodium : potassium ratio, and pulse were positive predictors.	Age, temperature, BMI, waist circumference, hip circumference, nonmigration status, number of children, sodium : potassium ratio, and ambient temperature.	Linear regression modelling

Bank workers [44]	Ibadan	BP correlated with income and education in men but not in women, after adjusting for age and BMI.						Higher income; educational level variable between men and women after adjusting for age and BMI.			Multiple linear regression; MANOVA

Oil, printing, cotton mill, tobacco factory, canning factory, and hotel workers [38]	Dakar	SBP was associated with age, BMI, higher educational level, tea consumption, and ethnicity in men. DBP associated with age and BMI in men. In women, age and BMI were the only significant factors associated with both SBP and DBP.	Age statistically significant association with SBP and DBP in men and women.			BMI statistically significant association with both SBP and DBP in women and with DBP but not SBP in men.		Educational level with illiterates having highest levels of BP is associated with SBP but not DBP.	Tea consumption associated with SBP.	Age, BMI, shift work, tea consumption, occupational category, educational level, and ethnicity.	Multiple linear regression

BMI: body mass index; DBP: diastolic blood pressure; SBP: systolic blood pressure; SES: socioeconomic status.

**Table 3:** Determinants of hypertension in workers in West Africa.

Study population	Location	Determinants	Age	Sex	Physical activity	Obesity or adiposity	Alcohol	SES	Others	Variables in model	Type of analysis
Civil servants [11]	Benin City	BMI (OR 2.88 Tertile 3 versus Tertile 1), age (OR 10-year groups 1.42), alcohol drinking (OR 1.85), and high SES (OR 2.62) were all independent risk factors for HTN in men but not education. Only BMI (OR 13.2) related to HTN in women.	Age [OR 10-year groups 1.42, 95% CI 1.03–1.96] in men; not significant in women	—	—	BMI [OR 2.88 Tertile 3 versus Tertile 1, 95% CI 1.6–5.1 in men]; OR 13.2, 95% CI 1.5–113.0 in women	Alcohol drinking (OR 1.85, 95% CI 1.15–2.98) in men; not significant in women	Senior staff in men (OR 2.62, 95% CI 1.61–4.29); not significant in women	—	Staff grade, BMI, alcohol drinking, and age in 10-year intervals.	Logistic regression

Civil servants [10]	Sokoto	Only age group and BMI tertile in men and sodium excretion were statistically significant. No significant association with sodium excretion or SES in men or with age, BMI, or staff grade in women.	10-year age intervals OR = 2.4 (95% CI 1.8–3.4) in men			Tertile 3 OR = 2.9 (1.6–5.1) in men; OR = 13.2 (1.5–113.0) in women	Alcohol drinker OR = 1.9 (1.2–3.0) in men	Senior staff OR = 2.6 (1.6–4.3) in men	Potassium excretion not significant	Age group 10-year groups, BMI, sodium excretion, and staff status.	Logistic regression

Civil servants [18]	Ibadan	For both sexes, normalized WHR, plasma glucose, age, and family history of diabetes. Only age predicted HTN in women.	OR = 1.1	NS	—	WHR, OR = 1.35	—	—	Height not associated with BP or HTN	Age, family history of diabetes, sex, plasma glucose, and normalized WHR.	Logistic regression

Civil servants [9]	Accra	Age group > 35 years, male sex, and physical inactivity	Age group 35–44 years OR = 3.8 (2.0–7.4); 45–54 years OR = 11.3 (6.4–21.5); ≥55 years OR = 15.0 (8.2–29.8)	Female sex OR = 0.71 (0.50–0.99)	Moderate OR = 0.7 (0.5–1.0); intense OR = 0.6 (0.3–1.2)	NS	NS			Sex, age groups, BMI, physical activity, and alcohol use.	Logistic regression

Civil servants [65]	Accra	Positive graded association between staff grade and HTN and current wealth when adjusted for age and sex. Statistically significant associations are lost when BMI is controlled for.						NS		Age, sex, preadult and current wealth, employment grade, level of education, and BMI.	Logistic regression with age as continuous variable

Civil servants [13]	Kaduna City	Age group ≥ 40 years, family history (OR 1.5, 95% CI 1.1–2.1).	AOR ≥ 40 years = 6.7 (95% CI 4.1–11.0) compared with age group < 40 years						Marital status not significantly associated with HTN	Age, marital status, and family history	Logistic regression

Health workers in a Teaching Hospital [27]	Jos City	Alcohol (OR 2.58, *p* = 0.03) and obesity (OR 3.37, *p* = 0.006) were found to be independently associated with hypertension.				OR = 3.37, *p* = 0.006	OR = 2.58, *p* = 0.03			Age, sex, BMI, and alcohol	Logistic regression

Health workers [31]	Yenagoa	Age groups > 30 years.	AOR 30–45 years = 4.147 (95% CI 1.25–13.67), > 45 years = 5.45 (95% CI 1.46–22.80) compared with age group < 30 years	NS	—	Truncal obesity (WC > 102 cm in men, >88 cm in females) versus normal WC; AOR = 3.64 (95% CI 1.15–9.06). BMI and WHR = NS	—	Educational level: NS	Marital status: NS	Age, sex, marital status, level of education, family history of HTN, past history of diabetes, family history of diabetes, history of alcohol use, smoking, BMI, WC, and WHR.	Logistic regression

Male factory workers [39]	Ibadan	Education associated with BP, after adjusting for age, BMI, pulse, and alcohol consumption.								Age, BMI, pulse rate, and current alcohol drinking.	Multiple logistic analysis of covariance

Market workers (traders and artisans) [36]	Enugu	Age, BMI, and alcohol consumption predicted HTN.	Increasing age	Not significantly associated with HTN		Significant factor	Significant factor	—		Age, sex, smoking, snuff tobacco, alcohol, BMI, WHR, and educational status.	Multiple linear regression

HTN: hypertension; OR: odds ratio; SES: socioeconomic status.
